# Interleukin-1 Family Cytokines in Liver Diseases

**DOI:** 10.1155/2015/630265

**Published:** 2015-10-15

**Authors:** Hiroko Tsutsui, Xianbin Cai, Shuhei Hayashi

**Affiliations:** Department of Microbiology and Department of Pu-Erh Tea and Medical Science, Hyogo College of Medicine, 1-1 Mukogawa-cho, Nishinomiya 663-8501, Japan

## Abstract

The gene encoding IL-1 was sequenced more than 30 years ago, and many related cytokines, such as IL-18, IL-33, IL-36, IL-37, IL-38, IL-1 receptor antagonist (IL-1Ra), and IL-36Ra, have since been identified. IL-1 is a potent proinflammatory cytokine and is involved in various inflammatory diseases. Other IL-1 family ligands are critical for the development of diverse diseases, including inflammatory and allergic diseases. Only IL-1Ra possesses the leader peptide required for secretion from cells, and many ligands require posttranslational processing for activation. Some require inflammasome-mediated processing for activation and release, whereas others serve as alarmins and are released following cell membrane rupture, for example, by pyroptosis or necroptosis. Thus, each ligand has the proper molecular process to exert its own biological functions. In this review, we will give a brief introduction to the IL-1 family cytokines and discuss their pivotal roles in the development of various liver diseases in association with immune responses. For example, an excess of IL-33 causes liver fibrosis in mice via activation and expansion of group 2 innate lymphoid cells to produce type 2 cytokines, resulting in cell conversion into pro-fibrotic M2 macrophages. Finally, we will discuss the importance of IL-1 family cytokine-mediated molecular and cellular networks in the development of acute and chronic liver diseases.

## 1. Introduction

The supernatants of activated leukocytes were shown to have the capacity to activate lymphocytes in the 1940s [1]. In 1979, four decades later, the active molecule was designated as interleukin- (IL-) 1. In 1984, the cDNA for murine IL-1 was identified. IL-1 is composed of two different molecules, IL-1*α* and IL-1*β*, which are recognized by the same receptor (R), known as the IL-1 receptor, which consists of IL-1R1 and IL-1RAcP [1]. Both IL-1*α* and IL-1*β* play roles in innate immunity. Subsequently, a third molecule, IL-1R antagonist (IL-1Ra), was discovered [2]. IL-1Ra competitively binds to IL-1R1 but does not have the capacity to activate IL-1 signaling. In 1995, another member of the IL-1 family, interferon-*γ* inducing factor (IGIF), was identified [3]. Due to its homology with IL-1*α* and IL-1*β* and to the similarity of its mode of production to that of IL-1*β*, IGIF was temporarily called “IL-1*γ*” [4]. Indeed, like IL-1*β*, IGIF lacks a leader peptide and is produced as a biologically inactive precursor (pro). Pro-IGIF, like pro-IL-1*β*, requires posttranslational processing for activation and secretion [5]. However, IGIF has been shown to require IL-18R but not IL-1R for signaling transduction; neither IL-1*α* nor IL-1*β* requires IL-18R [6]. This indicates that IGIF is a member of the IL-1 family but not of IL-1 itself. Thus, IGIF was designated as IL-18. IL-18 is a unique cytokine that activates both innate and acquired immunity, including both Th1 and Th2 immune responses, and has important pathophysiological functions. In 2005, another member of the IL-1 family, IL-33, was identified [7]. Unlike IL-1 or IL-18, IL-33 is important for the development of the type 2 immune response and several diseases. Subsequently, several proteins that show homology with IL-1 family members but have different biological functions have been discovered, such as IL-36*α*, IL-36*β*, IL-36*γ*, IL-36Ra, IL-37, and IL-38 [8]. In this review, we will focus on the roles of IL-1, IL-18, and IL-33 in various liver diseases, following a brief introduction to the IL-1 family, with the aim of clarifying the molecular and cellular networks involving each cytokine that are activated in liver diseases. We will focus on the unique mechanism of liberation of each cytokine and on the liver resident cells that may release and respond to each cytokine. Many excellent reviews have been published that can provide the reader with recent, detailed histological findings on the members of the IL-1 family [9–11].

## 2. The IL-1 and IL-1R Families

**Figure 1:**
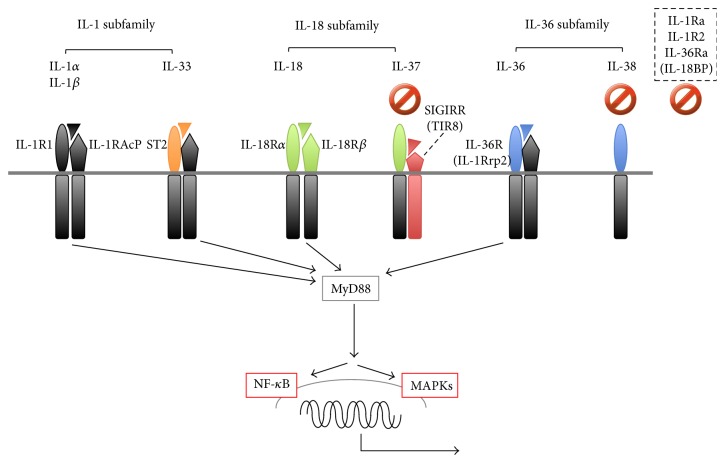
IL-1 family agonists and antagonists. The IL-1 family is divided into three subfamilies based on the length of the N-terminal pro-pieces. The IL-1 subfamily consists of IL-1*α*, IL-*β*, IL-1 receptor antagonist (IL-1Ra), and IL-33. The IL-18 subfamily is composed of IL-18 and IL-37. IL-36*α*, IL-36*β*, IL-36*γ*, and IL-38 belong to the IL-36 subfamily. The receptor for each IL-1 family cytokine is a heterodimer of the proper or common subunits. IL-1R1, ST2, IL-18R*α*, and IL-36R are ligand-binding subunits, whereas IL-1R accessory protein (IL-1LRAcP), IL-18R*β*, and SIGIRR (TIR8) are signaling subunits. Upon engagement of the binding subunits with the corresponding ligands, they recruit the corresponding signaling receptor subunit, which, except in IL-37 signaling, ultimately translocates nuclear factor- (NF-) *κ*B to the nucleus and activates MAPKs such as p38 and JNK. IL-38, IL-1Ra, IL-18-binding protein (IL-18BP), and IL-36Ra inhibit IL-36, IL-1, IL-18, and IL-36 signaling, respectively. IL-37 inhibits the signal pathways of the innate and acquired immune responses via mechanisms that are poorly identified. IL-1R2 acts as a decoy receptor for IL-1*α* and IL-1*β*. Stop signs indicate proteins that inhibit corresponding ligand signaling or those that negatively regulate other signal pathways as well. JNK: c-jun-N-terminal kinase; MAPK: mitogen-activated protein kinase; SIGIRR: single immunoglobulin IL-1-related receptor; ST2: suppression of tumorigenicity 2.

The IL-1 family ligands and the corresponding receptor subunits are shown in Figure 1. All of the receptor subunits are members of the IL-1R family and are characterized by possession of a Toll-like receptor (TLR)/IL-1R (TIR) domain within their cytoplasmic tails. TIR is required by IL-1 family members and TLR ligands for signaling. IL-1 family ligands comprise seven members with agonistic functions (IL-1*α*, IL-1*β*, IL-18, IL-33, IL-36*α*, IL-36*β*, and IL-36*γ*) and four members with antagonistic activities, such as IL-1Ra, IL-36Ra, IL-37, and IL-38. IL-1R family members include four ligand-binding subunits, IL-1R1, ST2, IL-18R*α*, and IL-36R, and three signaling subunits, IL-1RAcP, IL-18R*β*, and single immunoglobulin IL-1-related receptor (SIGIRR), alternatively named TIR8. SIGIRR/TIR8 is regarded as an orphan receptor with regulatory functions [12].

IL-1 family ligands are classified into three groups, based on the size of the N-terminal pro-pieces that remain after cleavage of the full-length ligands [9–11]. Members of the IL-1 subfamily, such as IL-1*α*, IL-1*β*, and IL-33, possess the longest pro-pieces, composed of approximately 270 amino acids. Both IL-1*α* and IL-1*β* transduce signals via IL-1R, a heterodimer of IL-1R1 and IL-1RAcP. IL-33 uses ST2 and IL-1RAcP. IL-18 subfamily ligands, including IL-18 and IL-37, also possess long pro-pieces composed of approximately 190 amino acids. The receptor for IL-18 consists of a binding subunit, IL-18R*α*, and a signaling chain, IL-18R*β*. IL-37 binds to IL-18R*α*, and SIGIRR (TIR8) is required as the signaling subunit. Notably, the mouse homologue of IL-37 remains unknown. IL-36 subfamily ligands such as IL-36*α*, IL-36*β*, IL-36*γ*, and IL-38 possess small pro-pieces of approximately 150 amino acids. All except IL-38 bind to IL-36R, followed by signaling via the coreceptor, IL-1RAcP.

The biological functions of IL-1 (IL-1*α* and IL-1*β*) and IL-36 (IL-36*α*, IL-36*β*, and IL-36*γ*) are negatively regulated by IL-1Ra and IL-36Ra, respectively. IL-1Ra, in competition with IL-1*α* and IL-1*β*, binds to IL-1R1, but unlike the IL-1*α*/IL-1R1 or IL-1*β*/IL-R1 complexes, the IL-1Ra/IL-1R1 complex cannot recruit the signaling chain, IL-1RAcP, eventually resulting in inhibition of IL-1 signaling [13]. Similarly, IL-36Ra binds to IL-36R and inhibits recruitment of the IL-36 signaling receptor chain, IL-1RAcP, onto IL-36R [14]. IL-1R2 is a “decoy receptor” that competitively inhibits IL-1*α*/*β* binding to IL-1R1. IL-18-binding protein (IL-18BP), which does not belong to the IL-1 family, is a natural inhibitor of IL-18 [15].

Following binding of a ligand to its corresponding binding receptor subunit, the ligand/binding receptor subunit complex recruits the corresponding signaling subunit. In this setting, the cytoplasmic tails of the binding and signaling subunits can recruit and interact with the signaling adaptor molecule MyD88 via TIR-TIR interaction, which eventually results in the nuclear translocation of nuclear factor- (NF-) *κ*B and the activation of mitogen-activated protein kinases (MAPKs) such as p38 and c-jun-N-terminal kinase (JNK) [14, 16, 17] (Figure 1).

## 3. Modes of Liberation of Biologically Active Ligands

**Figure 2:**
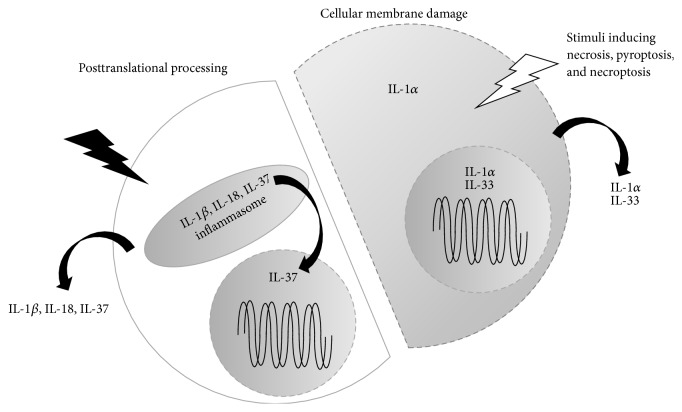
Modes of action of IL-1 family cytokines. All cytokines belonging to the IL-1 family, except IL-1 receptor antagonist (IL-1Ra), lack the leading peptides that are required for cell secretion. Furthermore, all full-length cytokines, except IL-1*α* and IL-33, are biologically inactive unless they receive appropriate posttranslational processing. Even biologically active full-length IL-1*α* and IL-33 need the appropriate cellular stimuli to be secreted from cells. IL-33 is localized in cellular nuclei, whereas IL-1*α* is localized in lysosomes and perhaps in nuclei. After receiving stimuli that induce cell death via destruction of the cellular membranes (i.e., pyroptosis and necroptosis), IL-1*α* and IL-33 are extracellularly liberated. In contrast, precursor- (pro-) IL-1*β*, pro-IL-18, and pro-IL-37 require cleavage by caspase-1 in the inflammasome, a large multiple-protein complex (shown in Figure 3). Following appropriate stimuli, the inflammasomes are activated. Consequently, biologically active IL-1*β*, IL-18, and perhaps IL-37 fragments are secreted. Bioactive IL-37 can also be translocated into nuclei. IL-36 subfamily members also require posttranslational processing for activation and excretion. However, they cannot be processed by caspase-1; their processing enzymes remain to be elucidated. pro: precursor.

Because they lack leader peptides, none of the members of the IL-1 family, except IL-1Ra, are extracellularly released immediately after translation. The full-length cytokines remain inside the cell, unless the cell is appropriately stimulated or damaged. Some full-length ligands, such as IL-33 and IL-1*α*, are biologically active but require cellular damage for release [18–20] (Figure 2 right). Full-length IL-1*α* is biologically active, but processed IL-1*α* has been reported to exert higher activity than full-length IL-1*α* [21, 22]. Full-length IL-1*α* and IL-33 are not released unless the cellular membrane is destroyed. Thus, IL-1*α* and IL-33 are designated as alarmins.

Some of the IL-1 family members are produced as biologically inactive precursor proteins and require appropriate posttranslational processing for activation and release from cells, such as IL-1*β*, IL-18, IL-37, IL-36*α*, IL-36*β*, IL-36*γ*, and IL-36Ra. IL-1*β*, IL-18, and IL-37 are processed by caspase-1, which was initially termed IL-1*β*-converting enzyme (ICE) [5, 18, 23–25] (Figure 2 left). In contrast, IL-36 subfamily members commonly lack caspase-1 cleavage sites. Their processing enzyme remains to be identified [14].

**Figure 3:**
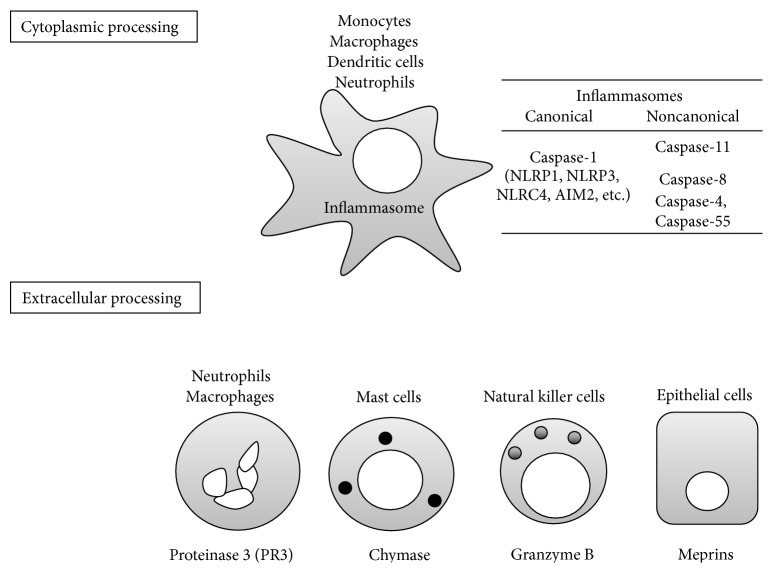
Processing enzymes dependent on and independent of the inflammasome. Caspase-1 is an enzyme that putatively converts IL-1*β* and IL-18. Caspase-1 is produced as zymogen and requires cleavage. Inflammasomes are the machinery in which caspase-1, IL-1*β*, and IL-18 are processed. Inflammasomes are classified as canonical and noncanonical. Canonical inflammasomes can be divided into 4 main types based on differences in cytoplasmic PRRs, such as NLRP1, NLRP3, NLRC4, and AIM. Noncanonical inflammasomes activate caspase-11, caspase-8, caspase-4, and caspase-5 and often collaborate with the canonical inflammasomes. Monocyte-macrophage lineage cells, dendritic cells, and neutrophils can harbor inflammasomes. Various enzymes have the capacity to process IL-1*β* and IL-18 extracellularly. Neutrophil-derived proteinase 3, mast cell chymase, granzyme B produced by NK cells, and meprins produced by epithelial cells can convert pro-IL-1*β* and pro-IL-18 into mature ligands. AIM: absent in melanoma 2; NK: natural killer; NLRC: NACHT, leucine-rich repeat, and caspase recruitment domain-containing protein; NLRP: NACHT, leucine-rich repeat, and pyrin domain-containing protein; PRR: pattern recognition receptor.

Caspase-1 is a member of the caspase family [26]. Caspase family members are produced as enzymatically inactive zymogens. Based on function, caspases have been classified into two groups, cell death-associated caspases (caspase-8, caspase-9, caspase-6, caspase-7, and caspase-3) and inflammatory caspases (caspase-1, caspase-4 caspase-5, and caspase-11). The activation cascade of the cell death-associated caspases was identified in the 1990s. However, the molecular mechanism for the activation of inflammatory caspases remained enigmatic until 2002. The late Dr. J. Tschopp proposed that a large protein complex, named the inflammasome, was the cytoplasmic machinery that activates caspase-1 for release of resultant mature IL-1*β* and IL-18 [27]. Several types of inflammasomes, with different cytoplasmic sensors, such as NACHT, leucine-rich repeat (LRR), and pyrin domain- (PYD-) containing protein- (NLRP-) 1 (NLRP1), NLRP3, caspase recruitment domain- (CARD-) containing protein- (NLRC-) 4 (NLRC4), and absent in melanoma 2 (AIM2) have since been identified (Figure 3 upper). Those proteins are cytoplasmic pattern recognition receptors (PRRs) that sense extrinsic pathogen-associated molecular patterns (PAMPs) and intrinsic damage-associated molecular patterns (DAMPs). Three of the proteins, NLRP1, NLRP3, and NLRC4, belong to the Nod-like receptor (NLR) family. AIM2 is a cytoplasmic DNA sensor that does not belong to the NLR family. When cells such as neutrophils, macrophages, or dendritic cells are appropriately stimulated [28], one of these sensors, a caspase-1 adaptor protein named apoptosis-associated speck-like protein containing a carboxy-terminal CARD (ASC) and procaspase-1, begins to assemble to form the inflammasome [29–33]. Structural analysis has revealed that, in some inflammasomes, such as NLRP3 and AIM2 inflammasomes, many procaspase-1 proteins likely polymerize to form a filamentous structure that permits autocleavage of procaspase-1 [34]. We found that the NLRP3 inflammasome is essential for the elevated serum levels of IL-18 observed in heat-killed* Propionibacterium acnes*-primed mice after challenge with lipopolysaccharide (LPS) [35]. Intriguingly, deletion of the genes encoding either component of the NLRP3 inflammasome or the resulting IL-18 prevented LPS-induced liver injury in* P. acnes*-primed mice. Indeed, *Nlrp*3^−/−^, *Asc* ^−/−^, *Caspase*1^−/−^, and *Il*18^−/−^ mice are resistant to liver injury induced by sequential treatment with* P. acnes* and LPS [3, 35, 36]. Recently, it was shown that IL-37 requires the NLRP3 inflammasome for activation and nuclear translocation [24]. In addition, several different types of noncanonical inflammasomes have been demonstrated to be required for activation of the canonical inflammasomes. For example, the caspase-11 noncanonical inflammasome was demonstrated to be essential for the activation of the canonical NLRP3 inflammasome in macrophages after Gram-negative bacterial infection [37–39]. Notably, ES cells derived from mouse strain 129 express little caspase-11, and the *Caspase*1^−/−^ mice currently used worldwide were generated from ES cells expressing* Caspase11* ^129/129^. Even after heavy backcrossing with C57BL/6 (B6) mice, *Caspase*1^−/−^ B6 mice still possess* Caspase11* ^129/129^, because of the close proximity of* Caspase1* and* Caspase11*. Therefore, data obtained using these *Caspase*1^−/−^ *Caspase*11^129/129^ mice are equivalent to data for *Caspase*1^−/−^ *Caspase*11^−/−^ mice. Before the function of endogenous caspase-1 could be determined, B6 ES cell-derived *Caspase*1^−/−^ mice had to be developed. Previous to the development of *Caspase*1^−/−^ B6 mice, we believed that caspase-1 was responsible for both release of IL-1*β*/IL-18 and pyroptosis, a type of regulated cell death accompanied by cell membrane rapture and resulting amplifying inflammatory responses, based on the observation that *Caspase*1^−/−^ *Caspase*11^129/129^ B6 macrophages (Mø) impaired both phenotypes. Finally, using *Caspase*11^−/−^ B6 mice, Kayagaki et al. demonstrated that caspase-11 participates in activation of the canonical inflammasome and is also responsible for pyroptosis. In contrast, *Caspase*1^−/−^ B6 mice did not show IL-1*β* and IL-18 release but did show pyroptosis comparable to wild-type (WT) B6 mice [37–40]. We now know that both noncanonical inflammasomes, involving caspase-11, and canonical inflammasomes, involving caspase-1, are required for IL-1*β*/IL-18 release, but that the canonical inflammasome is not required for pyroptosis upon infection with intracellular-facultative Gram-negative bacterial infection or LPS challenge. Kayagaki et al. clearly demonstrated that caspase-11-dependent pyroptotic cell death, but not caspase-1-mediated IL-1*β*/IL-18 release, is responsible for the morbidity and mortality of LPS-induced septic shock. Very recently, two human caspase-11 orthologues, caspase-4 and caspase-5, as well as caspase-11, were verified to directly recognize cytoplasmic LPS to activate the canonical inflammasome [41]. Furthermore, a noncanonical inflammasome involving caspase-8, an apoptosis-associated caspase, was also verified to be essential for processing IL-1*β* and IL-18 after microbial infection [42–46]. Thus, activation of canonical and noncanonical inflammasomes might result not only in release of IL-1*β* and IL-18 but also in activation of regulated cell death processes such as pyroptosis and apoptosis, which are characterized by the presence and absence of cell membrane rupture, respectively [47–50]. Thus, caspase family members are important for host defense via development of inflammatory responses and activation of regulated cell death in infected cells. Recent comprehensive reviews, including a description of the mechanisms for negative regulation of the inflammasome, provide detailed information on the inflammasome [47, 48, 51–57].

## 4. Extracellular Processing of Precursor Cytokines

Precursor cytokines can be activated by machinery other than inflammasomes. Many enzymes other than caspases can cleave pro-IL-1*β* and pro-IL-18 into biologically active fragments (Figure 3 lower).

Serine proteases can process IL-1*β* and IL-18. Neutrophils possess several types of serine proteases in their azurophilic granules. Neutrophil elastase, proteinase 3 (PR3), and cathepsin G are representative of neutrophil serine proteases [58, 59]. Purified PR3 activates the human Mø cell line THP-1 to release IL-1*β* independent of caspase-1 [60]. The importance of PR3-mediated IL-1*β* processing has also been demonstrated in vivo. WT mice, but not *Il*1*β* ^−/−^ mice, developed chronic arthritis after multiple intra-articular challenges with streptococcal cell wall. *Dppi* ^−/−^ mice, which lack the ability to activate PR3, are partly resistant to this chronic arthritis and show reduced induction of biologically active IL-1*β* in synovial explants, whereas *Caspase*1^−/−^ mice are similarly susceptible to WT mice [61]. The importance of PR3-mediated IL-1*β* processing has also been demonstrated in peritonitis induced by intraperitoneal challenge with monosodium urate crystals, a self-derived molecule relevant to gout [62]. Thus, multiple IL-1*β*-converting enzymes, including PR3, are required for inflammatory arthritis in mice [63]. PR3 is also involved in the processing of IL-18 and perhaps IL-33 [64, 65].

Human mast cell chymase was demonstrated to convert pro-IL-18 into biologically active IL-18 fragments [66]. Cutaneous mastocytosis is a common feature of atopic dermatitis in humans and also in murine models, such as skin-specific caspase-1 transgenic (tg) mice and IL-33 tg mice [67, 68]. In these mouse models, chronic dermatitis spontaneously develops with severe mastocytosis in the skin lesions. As IL-18 and IL-33 are potent activators of mast cells [69, 70], it is plausible that mast cells in the lesion release chymase. Chymase might, in turn, cleave pro-IL-18, which is liberated from the damaged keratinocytes, into active fragments. This might serve as a positive feedback circuit for exacerbation of chronic dermatitis in either mouse model.

Granzyme B is a cysteine protease, produced by natural killer cells and cytotoxic T lymphocytes, that is involved in apoptosis of target cells [71]. Recently, granzyme B was shown to exert processing activity on pro-IL-18 [72], although the biological settings involving granzyme B-mediated IL-18 processing are unclear.

As described below, many IL-1 family cytokines are produced as full-length proteins in nonhematopoietic cells, including epithelial cells, fibroblasts, and endothelial cells (Table 1). Membrane-bound and secreted astacin metalloproteinases, meprins, were verified to have the potential to cleave pro-IL-1*β* and pro-IL-18 into bioactive fragments [73, 74]. Meprin *α* is reported to be expressed in the stratum basale of human skin, whereas meprin *β* is observed in the cells of the stratum granulosum. Intriguingly, in the lesions of patients with psoriasis vulgaris, meprin *α* expression is predominant in the uppermost layers rather than the basal layer [75, 76]. This indicates that meprins may be involved in processing of IL-36 subfamily cytokines, which are preferentially expressed in the skin (as described above). Meprin *β*, but not meprin *α*, has been shown to be essential for IL-18 processing in a murine model of colitis induced by dextran sulfate sodium (DSS). After challenge with DSS, meprin *β*-deficient mice, but not meprin *α*-deficient mice, display a significant reduction in the elevation of serum levels of IL-18 in comparison with WT-mice [74]. Thus, in nonhematopoietic cells, meprins are IL-1*β*- and IL-18-converting enzymes.

Previously, inflammasome-independent enzymes were believed to be involved in the processing of IL-1*β* in autoinflammatory disease of *Pstpip*2^cmo^ mice [77–81]. *Pstpip*2^cmo^ mice express a Leu98Pro missense mutation in the Pombe Cdc15 homology family protein proline serine-threonine phosphatase interacting protein 2 (PSTPIP2) and develop osteomyelitis when they are fed a normal, low-fat diet. *Pstpip*2^cmo^ *Il*1*β* ^−/−^ and *Pstpip*2^cmo^ *Il*1*r*1^−/−^ mice are free of osteomyelitis, whereas *Pstpip*2^cmo^ *Nlrp*3^−/−^ and *Pstpip*2^cmo^ *Casp*1^−/−^ mice suffer similarly to *Pstpip*2^cmo^ mice [77, 78]. Very recently, osteomyelitis was reported to be absent in *Pstpip*2^cmo^ mice fed a high-fat diet [82]. Intriguingly, in contrast to control normal mice, the microbiota of *Pstpip*2^cmo^ mice fed a low-fat diet was found to show a predominance of inflammation-associated commensals. In contrast, when these mice are fed a high-fat diet (HFD), their microbiota is restored. Furthermore, *Pstpip*2^cmo^ mice can evade osteomyelitis even when fed a low-chow diet, if the genes encoding caspase-1 and caspase-8 are both knocked out. However, the deletion of either* Caspase1* or* Caspase8 *alone does not prevent osteomyelitis. Thus, aberrant activation of either caspase-1 or caspase-8 seems to determine the severity of this autoimmune disease. The precise molecular mechanisms underlying this disease, in particular how caspase-1 and caspase-8 inflammasomes are involved, remain unclear.

Recently, a comprehensive review of inflammasome-independent regulation of IL-1 family cytokines was published [83]. It provides a detailed discussion of inflammasome-independent activation of IL-1 family cytokines.

## 5. Biological Functions

IL-1 family agonists signal via different corresponding receptor complexes, but commonly activate NF-*κ*B and MAPKs via recruitment of the signal adaptor MyD88 (Figure 1), resulting in the ability to perform their biological functions (Table 1). Recent findings on the function of each member will be briefly introduced.

### 5.1. IL-1

Innate immunity is required for the rapid eradication of invading microbes. However, the same innate immune response can be evoked upon exposure to stimuli from host-derived substances, such as PAMPs translocated from the gut microbiome or self-derived alarmins and DAMPs. This triggers or exacerbates inflammatory diseases. In contrast to inflammation induced by extrinsic microbial infection, this is known as sterile inflammation. Sterile inflammation underlies many types of local inflammatory diseases [84]. IL-1*α* has been demonstrated to play a major role in the development of sterile inflammatory diseases including atherosclerosis and myocardial infarction [85, 86]. Indeed, intraperitoneal administration of dead endothelial cell-derived particles can induce peritonitis in mice, dependent on IL-1*α* but not IL-1*β* [87]. Furthermore, it has been demonstrated that the mode of cell death largely determines whether cells release IL-1*α*, IL-1*β*, or neither [88]. Apoptosis and pyroptosis are two types of regulated cell death; necroptosis is a third. In contrast to apoptosis, pyroptosis and necroptosis are characterized by destruction of the cellular membrane. Apoptotic cells do not release their cellular contents, whereas both necroptotic and pyroptotic cells liberate their cellular contents, including IL-1*α* [48]. Pyroptosis, a type of inflammatory cell death induced by inflammasome activation as described above, is accompanied by IL-1*β* release. Necroptosis is mediated by receptor interacting protein kinase-3 (RIP3) and by its substrate, mixed lineage kinase like (MLKL), which is located in the cytosol [49, 89]. RIP3-phosphorylated MLKL is oligomerized and translocated to the plasma membrane, where it destroys the cell membrane by disruption of ion channels and/or induction of pore formation [90–93]. Necroptosis develops when cells are stimulated via cell death receptors, such as TNFR1, Fas, and receptors of TNF-related apoptosis-inducing ligand (TRAIL), under limited, apoptosis-inhibiting conditions. Other stimuli, such as TLR ligands, viral infection, and type 1 IFN, can induce necroptosis as well [49]. Unfortunately, no method has been established for selectively distinguishing necroptotic cell death from other types of cell death. Necroptosis can liberate IL-1*α*. In addition to IL-1*α*, IL-1*β* is responsible for sterile inflammation, because DAMPs in the liberated cellular contents can activate innate immune responses including inflammasome-mediated IL-1*β* release. Therefore, it is plausible that both IL-1*α* and IL-1*β* are involved in sterile inflammation [84].

Recent progress in antibody technology and tools for identification of disease-responsible genes has enabled cures for many patients with recurrent or chronic inflammatory diseases. In particular, many inflammatory diseases can be efficiently treated by IL-1 blockade. For example, patients with hereditary systemic autoinflammatory disease due to impaired regulation of inflammasome activation or impaired regulation of IL-1 signaling can be treated with an IL-1Ra named Anakinra and/or a soluble IL-1R named Rilonacept [13, 94–97]. Type 2 diabetes, to which the aberrantly activated inflammasome has been shown to be relevant [98–100], can also be treated with IL-1Ra [101].

### 5.2. IL-33

Analogous to IL-1*α*, IL-33 is liberated following cell death accompanied by cell membrane rupture (i.e., necroptosis but not apoptosis) [19].

Endogenous IL-33 is important for the type 2 immune response against helminth infection. Several cell types involved in innate immunity, such as group 2 innate lymphoid cells (ILC2), basophils, eosinophils, and mast cells, express IL-33R and play a role in triggering and/or enhancement of type 2 immunity. In particular, in response to IL-33, ILC2 promptly produces large amounts of IL-5 and IL-13, which eventually leads to eosinophilia and mucin accumulation around the helminth. IL-33-mediated ILC2 activation is important for the rapid development of Löffler syndrome, which is characterized by pulmonary eosinophilia after cutaneous infection of mice with intestinal nematode larva [102]. This type of ILC2-mediated host response is critical for the expulsion of intestinal nematodes via induction of parasite-specific type 2 immunity [103]. Thus, IL-33 activation of ILC2 is required for host defense against helminths via activation of both innate and acquired immune responses [104, 105].

Cerebral malaria, induced by infection with the protozoan* Plasmodium* spp., is a lethal disease in children. Th1 immunity against the protozoan is important for host defense but simultaneously activates collateral severe inflammation in the brain. Very recently, extrinsic IL-33 was demonstrated to protect against lethal cerebral malaria by activating ILC2, inducing regulatory T cells, and causing cellular conversion into M2 Mø that produce anti-inflammatory and regulatory cytokines [106].

IL-33 is pivotal for allergic diseases as well. ILC2 plays a role in the development of allergic airway inflammation [107–111]. For example, peripheral blood mononuclear cells (PBMCs), including ILC2, prepared from patients with allergic asthma produce larger quantities of IL-13 and IL-5 in response to IL-33 than those from control subjects [112]. In addition, the IL-33/IL-33R axis in mast cells and basophils participates in allergic respiratory diseases [113]. After intranasal infection of mice with a mouse parainfluenza virus, Sendai virus, chronic obstructive pulmonary disease (COPD) develops. The virus-infected WT mice show elevated airway resistance, whereas IL-33- and ST2-deficient mice do not [114]. The IL-33-IL-33R axis is also important for airway inflammation and for induction of IL-33 downstream cytokines and mucin. Notably, lung samples from patients with COPD exhibit higher levels of IL-33 in their lung epithelial cells than those from non-COPD patients [114]. IL-33 is essential for the development of allergic rhinitis induced by intranasal treatment with ragweed in mice [115]. This is also true for food allergies induced by topical application of peanut extracts [116]. Furthermore,* Il33* tg mice, which specifically overexpress IL-33 in their keratinocytes, spontaneously develop atopic dermatitis-like chronic cutaneous alterations [68].

Accumulating lines of evidence strongly suggest that IL-33 is involved in fibrotic diseases, an important cause of morbidity and mortality in humans. It is well established that type 2 immune responses involving cell conversion into M2 Mø are important for wound healing and fibrosis [117–120]. Because IL-33 is associated with type 2 immunity, as described above, it is plausible that IL-33 is causative of fibrotic diseases [121]. Serum levels of IL-33 are elevated in patients with systemic sclerosis, a connective tissue disease characterized by fibrosis of the skin and other organs, such as the lungs [122]. There is a positive relationship between serum IL-33 levels and disease severity [123]. In addition, daily subcutaneous injection of IL-33 induces cutaneous fibrosis in WT mice and mast cell-deficient mice (cKit^w/v^) but not in *St*2^−/−^, *Il*33^−/−^, *Rag*2^−/−^, or eosinophil-deficient mice (ΔdblGATA mice), indicating the importance of aberrant accumulation of IL-33, its downstream cytokine IL-13, acquired immunity, and eosinophils [124]. Large numbers of cells expressing abundant IL-33 are observed in the lungs of patients with idiopathic pulmonary fibrosis, as well as systemic sclerosis [125]. Furthermore, gene-delivery of* Il33* exacerbates bleomycin-induced pulmonary fibrosis [125, 126]. The roles of IL-33 in liver cirrhosis will be described in a subsequent section.

### 5.3. IL-18

IL-18 is a pleiotropic cytokine, similar to other IL-1 agonists, and its major biological functions have been described in several reviews [69, 127–130]. For example, IL-18 contributes to both type 1- and type 2-mediated host defense and inflammatory diseases. In collaboration with IL-12, IL-18 activates NK cells to produce a large quantity of interferon- (IFN-) *γ*. Convincingly, *Il*18^−/−^ mice are highly susceptible to infection with the intracellular bacterium* Listeria monocytogenes*, particularly in the early infectious phase [131]. IL-18 is also involved in the expulsion of the intracellular fungus* Cryptococcus neoformans*. In addition, together with IL-2, IL-18 activates NKT cells and T helper (Th) cells to produce the Th2-related cytokines, IL-4 and IL-13 [132]. IL-18 is known to be beneficial for the expulsion of the intestinal nematode,* Strongyloides venezuelensis* [133]. Unexpectedly, IL-18 activates Th1 cells to produce IL-13 and chemokines as well as Th1-related cytokines [134]. Based on this biological function, IL-18 can trigger the development of intrinsic atopic dermatitis and bronchial asthma, in which both IFN-*γ* and IL-13 play a critical role [67, 135, 136].

IL-18 has also been reported to be involved in metabolic syndrome [137]. In contrast to IL-1 [101], however, IL-18 protects against metabolic syndrome, although patients with the metabolic syndrome show increased serum levels of IL-18 [138, 139].

### 5.4. IL-37

In 2001, database analyses identified a new, IL-18R*α*-binding human protein named IL-37 [140]. To date, its mouse orthologue has not been identified. Various cell types have been reported to produce IL-37. IL-37 is a potent inhibitor of both innate and acquired immune responses.* IL37*tg mice are resistant to LPS-induced shock and DSS colitis compared to WT mice [141, 142]. IL-37 is associated with Behçet's disease, an intractable, chronic inflammatory disease [143]. IL-37 also dampens type 2 immune responses. Indeed, intranasal administration of human IL-37 ameliorates ovalbumin-induced type 2 allergic airway inflammation [144]. Upon IL-37 engagement, IL-18R*α* recruits SIGIRR/TIR8, which negatively regulates many signaling pathways, including phosphatase and tensin homolog deleted from chromosome 10 (PTEN) and signal transducers and activators of transcription- (STAT-) 3, to inhibit transforming growth factor *β* activated kinase- (TAK-) 1 and the transcription factors NF-*κ*B and MAPKs and to interact with Smad3, an anti-inflammatory signaling molecule [141, 145]. The full network that is activated by IL-37-mediated signaling remains to be elucidated.

### 5.5. IL-36

The IL-36 subfamily includes IL-36*α*, IL-36*β*, IL-36*γ*, IL-36Ra, and IL-38. All were discovered by database searches on the basis of their homology with IL-1*α* and IL-1*β* [8, 146]. IL-36 cytokines are expressed in the skin under normal conditions [147]. Skin-specific* Il36α* tg mice develop psoriasis-like skin alterations; they spontaneously suffer from flaky skin within 1 week of birth, but by week 3 their skin phenotypes have disappeared [148]. Thereafter, they exhibit recurrence of skin lesions. Treatment with anti-IL-23, anti-TNF, or anti-IL-36R antibodies rescues* Il36α* tg mice from the skin alterations [149]. Furthermore, following topical application of the ligand of TLR7/TLR8, imiquimod, *Il*36*ra* ^−/−^ mice develop more severe psoriasis-like skin alterations than WT mice. In contrast, *Il*23^−/−^ and *Il*17*a* ^−/−^ mice are resistant to imiquimod-induced psoriasis [150]. Several reports identified a homozygous missense mutation in* IL36RN* that encodes IL36Ra in patients with familial generalized pustular psoriasis and found that the mutations in* IL36RN* cause poor affinity or labile binding of IL-36Ra to IL-36R [151–153]. Thus, impaired IL-36Ra function might be responsible for the development of generalized pustular psoriasis. In addition, IL-36 agonists might be involved in sporadic psoriasis [154].

IL-38 is a recently identified IL-1 family antagonist that is reported to function similarly to IL-36Ra [155]. However, the biological roles of IL-38 remain to be elucidated.

## 6. The Roles of IL-1 Family Members in Liver Diseases

As mentioned, IL-1 family agonists and antagonists are involved in health and disease, and it is plausible that these molecules are involved in liver diseases. This section highlights recent advancements in the understanding of the molecular and cellular mechanisms of liver diseases associated with IL-1 family cytokines, particularly focusing on IL-1, IL-33, and IL-18.

### 6.1. IL-1 and IL-18 in Viral Hepatitis (Figure 4)

Because the viruses that cause liver diseases in humans are species-specific, it is difficult to demonstrate the mechanism of viral hepatitis in animal models other than primates, limiting advancement in the study of viral hepatitis. Study of immunodeficient mice with transplanted human liver cells and receiving human autologous hematopoietic stem cells may shed light on this issue. Mice with human liver and immune cells developed by induced pluripotent stem cells (iPS cells) show promise of becoming a useful tool in the near future.

#### 6.1.1. Hepatitis B Virus

It is well established that types I, II, and III IFNs directly inhibit the replication of hepatitis B virus (HBV) [156]. Recently, Watashi et al. demonstrated that IL-1 can protect against HBV infection [157]. The authors incubated hepatocytes with IL-1*β*, and after vigorous washing of the cells they added HBV to the cell culture. After 4 days of incubation they measured HBV replication. In this setting, IL-1*β* protected against HBV infection, whereas IFN-*α*, an HBV replication inhibitor, did not, suggesting that IL-1*β* inhibits the early phase of HBV infection, including attachment, entry, and nuclear import. This restriction of HBV infection is mediated by activation-induced cytidine deaminase (AID) induced by IL-1*β*/NF-*κ*B signaling. Indeed, introduction of AID suppresses the permission of HBV. Thus, IL-1*β* can restrict HBV infection via induction of AID, although the molecular mechanism underlying the AID-mediated restriction is unknown. Intriguingly, HBeAg, a soluble protein produced by HBV, can inhibit production of IFN-*γ* from IL-12/IL-18-activated NK cells via downregulation of IL-18/NF-*κ*B signaling [158]. Indeed, serum from HBeAg-positive patients induces lower IFN-*γ* production by IL-12/IL-18-stimulated NK cells than that from HBeAg-negative patients [158]. The liver contains abundant NK cells, which were initially named “pit cells” on the basis of their unique localization in the hepatic sinusoid [159], as well as Kupffer cells, which have the potential to produce both IL-18 and IL-12 in the same anatomical compartment. Thus, after microbial infection, robust IFN-*γ* is promptly produced by the intimate interaction between NK cells and Kupffer cells. HBV may evade this IFN-*γ* production by releasing HBeAg and can easily infect hepatocytes.

#### 6.1.2. Hepatitis C Virus

Type I and type III IFNs are important for eradication of HCV [160]. It is well documented that serum levels of IL-18 are elevated in chronic HCV patients. IL-18 levels reflect the severity and activity of HCV infection [161]. THP-1 cells or monocyte-derived Mø produce IL-1*β* and IL-18 after infection with HCV [162]. This HCV-induced IL-1*β*/IL-18 production requires activation of the TLR7/MyD88 pathway, but not TLR3/TRIF, and of NLRP3 inflammasomes. In contrast, IFN-*α* production requires mitochondrial antiviral-signaling protein (MAVS), which is an essential signal adaptor for the signaling of RIG-1-like receptors (RLRs), which are sensors of viral products. Thus, upon HCV infection, distinct modes of RRP signaling participate in production of IL-1*β*/IL-18 and IFN-*α* [163, 164].

### 6.2. The Importance of Necroptosis in IL-1*α*-Mediated Liver Diseases (Figure 4)

As described above, necroptosis is a form of regulated cell death that involves cellular membrane rupture. Necroptosis-driven liberation of the cell contents, including IL-1*α*, is important for the activation of sterile inflammation, which is a powerful mechanism for amplifying inflammation (described below). Hepatocyte necroptosis is involved in various liver diseases [165]. Acetaminophen (APAP) overdose is a predominant cause of acute, hepatotoxic liver failure in the USA and UK. An APAP-derived metabolite has been shown to have direct hepatocytotoxic action. Recent studies suggested that necroptotic hepatocyte death is a direct pharmaceutical effect of the APAP metabolite [166]. The liver of APAP-treated mice showed induction of RIP3, a key molecule that induces necroptosis, and RIP3 morpholin treatment protected against APAP-induced liver injury. In addition, *Rip*3^−/−^ hepatocytes are somewhat resistant to APAP. Very recently, Li et al. demonstrated that APAP-induced liver injury is induced by necroptosis of hepatocytes [167]. They verified that the B-Raf^v600E^ inhibitor dabrafenib, which is an anticancer drug, is a potent RIP3 inhibitor as well. Dabrafenib can inhibit necroptosis of human hepatocytes incubated with APAP. Importantly, inhibition of cell death by the RIP1 inhibitor necrostatin-1 had been regarded as a hallmark of necroptosis, but this inference is made dubious by the evidence of RIP1-independent necroptosis [49]. Thus, the necrostatin-1 test is not always useful for diagnosis of necroptosis. Similarly, necrostatin-1 treatment does not improve APAP-induced hepatocyte cell death [167]. Notably, positive staining for phosphorylated MLKL, a hallmark of necroptosis, has been observed in the hepatocytes of patients with APAP-induced liver failure [92]. Necroptosis has also been demonstrated to be relevant to ethanol-induced liver injury in mice [168]. This is also the case in liver biopsy of patients with alcoholic liver disease (ALD). Thus, some drug-induced liver injuries appear to develop in a manner dependent on RIP3-mediated necroptosis.

Ischemia-reperfusion injury of the kidney or retina was also demonstrated to be caused by necroptosis [169, 170]. Similarly, ischemia-reperfusion-induced liver injury might also involve the necroptotic death of hepatocytes. A precise analysis of whether and how necroptosis is involved in ischemia-reperfusion-induced liver injury would be useful.

RIP3 is overexpressed in the livers of patients with NASH [171]. In this paper, the authors showed that WT mice were susceptible to diet-induced NASH, whereas *Rip*3^−/−^ mice, which lack the capacity for necroptosis, were resistant. Therefore, necroptosis might underlie hepatocytic cell death during NASH. However, the endogenous factor(s) that are responsible for the initiation of RIP3-mediated necroptosis remain to be elucidated.

### 6.3. Sterile Inflammation Mediated by IL-1*α* and IL-1*β* (Figure 4)

Necroptotic hepatocytes in NASH, drug-induced liver injury, and possibly ischemia-reperfusion-induced liver injury can trigger sterile inflammation by activation of the NLRP3 inflammasome with liberated DAMPs and/or by IL-1*α*. It has been clearly demonstrated that saturated, but not unsaturated, free fatty acid (FFA), which is a host-derived metabolite, is capable of activating the NLRP3 inflammasome to produce IL-1*β* and that this IL-1*β* might lead to progression to NASH in mice with simple hepatic steatosis [172, 173]. The following scenario has been proposed. Saturated FFA can inhibit autophagy, especially mitophagy, in cells. Under normal conditions, impaired mitochondria are rapidly eliminated via mitophagy. However, saturated FFA-induced impairment of mitophagy might allow long-term retention of impaired mitochondria. Subsequently, aberrant production of reactive oxygen species (ROS) by the impaired mitochondria might continuously and robustly activate ROS-sensitive inflammasomes [172]. Miura et al. reported that the induction and exacerbation of NASH were explained by IL-1*β*, which is produced by the activation of TLR9/MyD88 signaling, and the NLRP3 inflammasome by DAMPs, possibly derived from necroptotic hepatocytes [174]. In fact, *Tlr*9^−/−^ mice are resistant to diet-induced NASH and show accompanying reduced induction of serum elevation of IL-1*β*. Consistently, *Myd*88^−/−^ and *Il*1*r*1^−/−^ mice show resistance to NASH similar to *Tlr*9^−/−^ mice. The authors reported that Kupffer cells were a major cellular source of IL-1*β*, which accelerated cytocidal lipid deposits in hepatocytes, induced cell death, and simultaneously activated hepatic stellate cells to produce profibrotic proteins. *Tlr*2^−/−^ mice evade NASH via impaired induction of IL-1*α* and IL-1*β*, even when fed a NASH-prone diet [175]. The importance of hematopoietic cells, including Kupffer cells, for the development of NASH via activation of TLR2 has been demonstrated in chimeric mice. Indeed, *Tlr*2^−/−^ Kupffer cells cannot produce IL-1*α* or IL-1*β* after stimulation with saturated free fatty acid palmitate together with TLR2 agonists. Sterile inflammation is also activated by hepatocyte-derived IL-1*β*. Hepatocytes prepared from mice suffering from NASH express enhanced levels of NLRP3 inflammasome components and have the capacity to release IL-1*β* in response to palmitate, a saturated fatty acid, in combination with LPS [176]. Consistently, treatment with pan-caspase inhibitors such as Emricasan and VX-166 can suppress liver injury, inflammation, and fibrosis in mice fed a NASH-prone, high-fat diet [177]. In contrast to IL-1, IL-18 is reported to be beneficial for preventing NASH. IL-18 secreted following activation of the NLRP3 inflammasome negatively regulates the development of murine NASH through maintenance of a healthy gut microbiota [178, 179]. This may imply that the harmful effects of IL-1*β* might overcome the beneficial action of endogenous IL-18.

Sterile inflammation is involved in the exacerbation of APAP-induced acute liver failure [180]. Imaeda et al. showed that treatment with a TLR9 antagonist and deletion of* Tlr9* decreased the severity of APAP-induced liver failure with impaired induction of* proIl1β*. NLRP3 inflammasome-deficient mice, such as *Caspase*1^−/−^, *Asc* ^−/−^, and *Nlrp*3^−/−^ mice, showed comparably reduced induction of liver failure to *Tlr*9^−/−^ mice. This suggests that DAMPs, including a TLR9 agonist, which are liberated from APAP-induced necroptotic hepatocytes, might activate the TLR9 pathway and the NLRP3 inflammasome.

The TLR9-mediated signal pathway has also been demonstrated to participate in the activation of sterile liver inflammation induced by ischemia and reperfusion [181]. A scenario similar to that of NASH and APAP-induced acute liver failure underlies the progression of this disease as well. An excellent review has been published that is helpful in understanding the other mechanisms underlying sterile inflammation in hepatic ischemia-reperfusion-induced liver injuries [182].

In the above-mentioned liver diseases, sterile inflammation may accelerate disease progress, such as lethal liver failure and fibrosis. However, the pathway that links TLR/MyD88-mediated pro-IL-1*β* production to activation of the NLRP3 inflammasome remains to be elucidated.

### 6.4. IL-33 in Liver Fibrosis (Figure 5)

Until recently, liver cirrhosis was regarded as the irreversible end stage of chronic liver disease. However, recent studies have revealed that liver fibrosis is reversible. Many cell types, such as Mø, pro-fibrotic myofibroblasts, which are mainly transdifferentiated from stellate cells, and innate and acquired immune lymphocytes, participate in the development of liver fibrosis. The mechanisms underlying the initiation of liver fibrosis have been gradually unraveled by intensive efforts using animal models and clinical data. For example, pro-fibrotic and pro-resolving Mø have been shown to contribute to the progression and regression of liver fibrosis, respectively [183, 184]. A comprehensive discussion of the cellular and molecular mechanisms of liver fibrosis discovered to date is out of the scope of this review. For further information, the reader may consult recent expert reviews [185–188]. Herein, we will focus on the roles of IL-33 in liver fibrosis.

The importance of IL-33 for liver fibrosis was demonstrated by analyses of several mouse models of liver fibrosis. Multiple injections of carbon tetrahydrochloride (CCl_4_) or thioacetamide, infection with the helminth* Schistosoma mansoni*, or bile duct ligation induces liver fibrosis in WT mice, accompanied by elevation of serum IL-33 levels [189, 190]. IL-33-expressing cells are accumulated in the fibrotic liver, particularly in *α*SMA^+^ cells [189, 190]. *Il*33^−/−^ mice are resistant to induction of liver fibrosis by these treatments. Intriguingly, multiple introduction of* Il33 *in the liver of mice induces liver fibrosis. *St*2^−/−^, *Il*13^−/−^, and *Il*4*rα* ^−/−^ mice are resistant to this treatment, strongly suggesting that the IL-33–IL-13 axis is important for the development of IL-33-induced liver fibrosis. *Rag*1^−/−^ mice lack both T- and B-lymphocytes but normally possess group 2 ILC (ILC2), which are capable of responding to IL-33 by prompt and robust production of type 2 cytokines including IL-13 [191–193]. Intriguingly, in vivo depletion of ILC2 prevents *Rag*1^−/−^ mice from developing IL-33-induced liver fibrosis. Thus, the IL-33–ICL2–IL-13 axis is critical for development of some types of liver fibrosis. Consistent with data from mice, serum levels of IL-33 are elevated in patients with liver cirrhosis [189]. In addition,* IL33* and* ST2 *expression levels in cirrhotic livers positively correlate with disease severity.

The IL-33–ILC2–IL-13 axis has also been demonstrated to be involved in biliary repair and possibly in carcinogenesis in the biliary tract. Biliary atresia is a bile duct disease that is common in childhood. A subset of patients with biliary atresia display an increase in pro-Th2 cytokines. Recently, it was demonstrated that the IL-33–ILC2–IL-13 axis plays a pivotal role in the development of biliary atresia-associated biliary repair and carcinogenesis [194, 195]. Serum IL-33 levels are elevated in patients with biliary atresia. Expression levels of* Il33* mRNA are correlated with numbers of replicating cholangiocytes in an experimentally induced mouse model of biliary atresia. Biliary atresia is induced by infection of neonate mice with rhesus rotavirus type A (RRP). Treatment with anti-ST2 antibodies inhibits cholangiocyte replication in RRP-challenged mice. Conversely, extrinsic IL-33 treatment induces profound replication of extrahepatic cholangiocytes. ILC2-deficient *Rag*2^−/−^ *γ*c^−/−^ mice, as well as *Il*13^−/−^ mice, fail to show cholangiocyte growth upon IL-33 treatment. Thus, the IL-33–ILC2–IL-13 axis is important for bile duct repair. Similarly, it was reported that IL-33 facilitated oncogene-induced cholangiocarcinoma in mice [196].

### 6.5. IL-33 in Obesity, a Precondition for NASH (Figure 6)

IL-33/ST2 signaling protects against obesity-associated inflammation in visceral adipose tissue (VAT) and maintains insulin sensitivity. It is well established that chronic, low-grade inflammation involving Mø in VAT links obesity to metabolic diseases [197, 198]. In parallel, regulatory T cells (Treg) are functionally and numerically reduced in the VAT of obese mice, which might partly explain the dysregulated inflammation observed in the VAT of obese mice [198, 199]. Multiple injections of IL-33 improved insulin sensitivity and glucose tolerance in* ob/ob* mice, concomitant with cellular conversion into anti-inflammatory M2 Mø in their VAT [200]. Consistent with this, *St*2^−/−^ mice were highly susceptible to HFD-induced impairment of glucose tolerance. IL-33 treatment could reverse the deficit of ST2^+^ Treg in diet-induced obese mice [201]. Furthermore, accumulation of Treg in the VAT of lean, aged mice was reported to be dependent on IL-33 in VAT. Indeed, the proportion of Treg in CD4^+^ T cells in the VAT of *St*2^−/−^ mice is reduced compared to that of WT mice. Conversely, IL-33 treatment increases the proportion of Treg [202, 203]. In addition, ILC2 is required for the accumulation of eosinophils and cell conversion to M2 Mø in the VAT via production of IL-5 and IL-13 [204].

White adipose tissue (WAT) stores energy. Unlike WAT, brown adipose tissue (BAT) contributes to energy consumption via thermogenesis and is regarded as a possible target organ for treatment of obesity. Although the VAT is lost by adulthood in humans, adult humans harbor beige adipocytes that express a thermogenic protein, uncoupling protein 1 (UCP1), in subcutaneous WAT (SAT) in cold conditions. Thus, beige fat shows promise as a therapeutic target for the treatment of obesity. It has been demonstrated that both IL-4 production by eosinophils and the IL-4 responsiveness of M2 macrophages are equally required for the development of thermogenic beige fat that expresses UCP1 [205]. Brestoff et al. and Lee et al. independently demonstrated that, in this setting, IL-33 activation of ILC2 is important for beiging [206, 207]. In obese humans, the SAT harbors lower numbers of ILC2 than in nonobese humans. This is also true for diet-induced obese WT mice. *Il*33^−/−^ mice harbor small numbers of ILC2 in the SAT and show higher gain of body and fat mass than WT mice, even when fed a normal, low-fat diet. The SAT of *Il*33^−/−^ mice is characterized by the presence of few beige adipocytes and by much lower expression of* Ucp1*, whereas in WT mice the SAT as well as the VAT harbors substantial numbers of beige adipocytes. IL-33 administration induces an increase in ICL2 in the SAT and enhances calorie expenditure in WT mice. Transfer of ILC2 prepared from IL-33-treated WT mouse SAT upregulates* Ucp1* expression in the SAT and increases energy expenditure in WT mice. Transfer of ILC2 induces SAT* Ucp1 *expression in ILC2-null *Rag*2^−/−^ *γc* ^−/−^ mice as well. Levels of proprotein convertase subtilisin/kexin type 1 (*Pcsk1*), an endopeptidase involved in processing prohormones into active form, were shown to increase in ILC2 [206]. Following administration of methionine-enkephalin peptide, which is a substrate of PCSK1, WT mice showed increased numbers of UCP1^+^ beige adipocytes and increased energy consumption. This may imply that IL-33 induces beiging in the WAT, at least partly via production of methionine-enkephalin peptide. Taken together, these results show that endogenous IL-33 sustains beige adipocytes in the SAT by activating ILC2, presumably in collaboration with M2 Mø and eosinophils, which eventually contributes to metabolic homeostasis by tuning calorie expenditure. Endogenous opioid-like peptides, including the methionine-enkephalin produced by IL-33-activated ILC2, show promise as a potent therapeutic agent for obesity and obesity-associated diseases.

## 7. Closing Remarks

Recent studies have revealed the importance of ILC2 in health and disease. ILC2 plays a critical role in the development of liver fibrosis and in protection against obesity. However, these conclusions were drawn from studies using mouse models, and although the definition of mouse ICL2 is well established, definitions of human ILC2 differ among research groups [208, 209]. A uniform definition of ICL2 will be required to make progress in the translation of the findings on murine ICL2 to human diseases.

**Figure 4:**
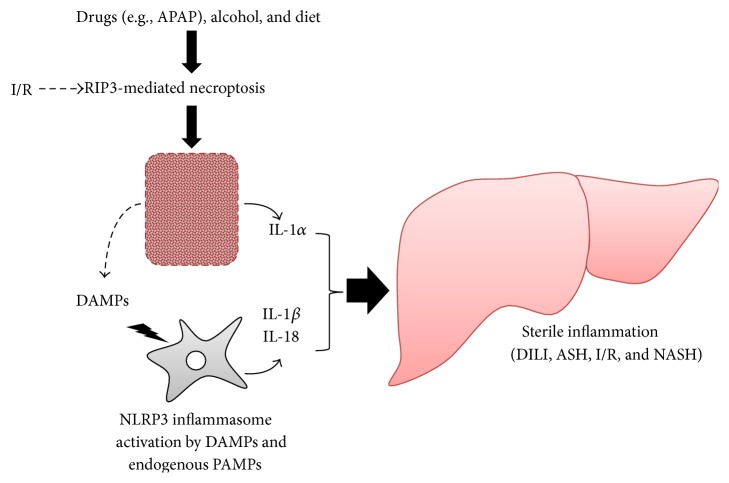
Mechanisms for IL-1-mediated liver diseases. Intake of acetaminophen, alcohol, or perhaps diet induces necroptosis of hepatocytes via activation of RIP3. Ischemia-reperfusion also causes RIP3-mediated hepatocyte necroptosis. Necroptotic hepatocytes release their cellular contents, including IL-1*α* and DAMPs. DAMPs such as saturated FFAs, which are metabolites of high-calorie diets, and endogenous PAMPs, such as LPS translocated from the gut microbiome, can activate NLRP3 inflammasomes to release IL-1*β*. In turn, IL-1*β* and IL-1*α* exacerbate the liver inflammation and injury. This inflammation-amplifying mechanism is known as “sterile inflammation,” given the absence of exogenous microbes or their PAMP products. In contrast, IL-18 regulates NASH via maintenance of healthy intestinal microbiota. APAP: acetaminophen; ASH: alcoholic induced steatohepatitis; DAMPs: damage-associated molecular patterns; DILI: drug-induced liver injury; I/R: ischemia-reperfusion injury; NASH: nonalcoholic steatohepatitis; NLRP3: NACHT, leucine-rich repeat, and pyrin domain-containing protein-3; PAMPs: pathogen-associated molecular patterns; RIP3: receptor interacting protein kinase-3.

NASH is characterized by liver fibrosis. Overexpression of IL-33 can induce liver fibrosis in mice. However, it remains to be elucidated whether endogenous IL-33 is involved in diet-induced NASH fibrosis. Many investigators have reported the elevation of IL-33 or IL-33 mRNA levels in the liver or in circulation. However, it remains unclear whether the IL-33 elevation is a result of or relevant to liver fibrosis. To address this, we must examine whether *Il*33^−/−^ and *St*2^−/−^ mice are resistant to liver fibrosis. In addition, the cellular source of IL-33 in these diseases remains unknown. Liver parenchymal cells have been shown to be a major cellular source of IL-33 in the liver under normal conditions. During the progression of NASH, *α*-SMA-expressing myofibroblasts become the predominant IL-33-producing cell type. However, this does not indicate whether myofibroblast-derived IL-33 initiates and/or exacerbates liver fibrosis, because we do not know whether or how the myofibroblasts undergo the necroptotic or pyroptotic cell death that allows liberation of intracellular IL-33. It is well documented that hepatocytes become necroptotic under NASH conditions (Figure 4), suggesting that hepatocytes might be a major continuous cellular source of IL-33 during NASH.

Obesity is an important problem in both developed and developing countries. Obesity plays a central role in the development of various life-threatening diseases, including cardiovascular diseases and cancers. However, the causes of the rapid, global increase in obesity remain to be elucidated. Recent studies clearly demonstrate that artificial sweeteners and dietary emulsifiers, which are a common part of the daily diet, are responsible for the development of metabolic syndrome in mice [210, 211]. Eating a diet without these agents might be important for health. In addition, several active compounds in a “healthy diet” have been identified. The biological compounds thymoquinone, curcumine, and resveratrol can be purified from* Nigella sativa* (black seed),* Zingiberaceae* spp. (e.g., ginger and turmeric), and grape skins, respectively [212–214]. These compounds are beneficial for human health. Many traditional foods have been shown to promote health. The identification of pro-health compounds will contribute to human health and longevity.

## Figures and Tables

**Figure 5:**
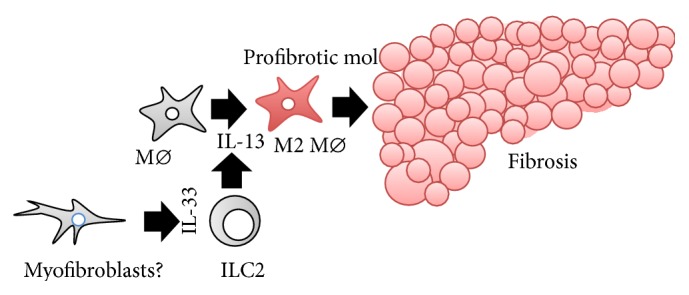
IL-33–ILC2–IL-13 axis-mediated liver fibrosis. The IL-33–ILC2–IL-13 axis is important for physiological wound healing. The same process is involved in the development of liver fibrosis. IL-33 activates ILC2 to release IL-13. In response to IL-13, liver macrophages undergo cell conversion into M2 Mø, which begin to produce profibrotic proteins, eventually leading to the development of liver fibrosis. Myofibroblasts are regarded as a major cellular source of IL-33 in cases of liver fibrosis. ILC2: group 2 innate lymphoid cells; Mø: macrophages.

**Figure 6:**
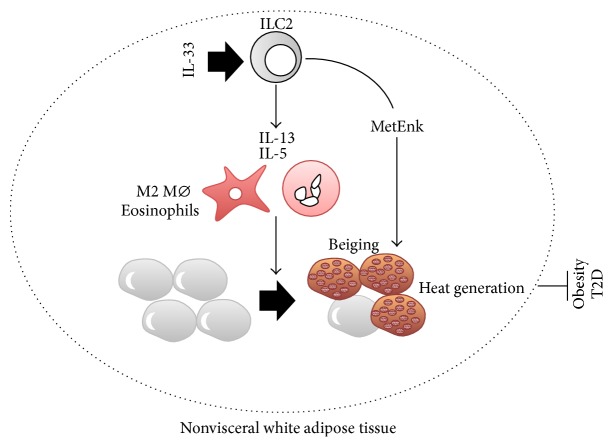
IL-33–ILC2 beiges white adipose tissue to protect against obesity via heat generation and energy expenditure. IL-33–ILC2–IL-13/IL-5 is also critical for transformation of white adipocytes into thermogenic beige adipocytes. In response to IL-33, ILC2 in white adipose tissue produces methionine-enkephalin peptide (MetEnk) as well as pro-allergic IL-13 and IL-5. IL-13 and IL-5 induce recruitment and activation of eosinophils and cell conversion of Mø into M2 Mø. MetEnk, combined with M2 Mø and eosinophils, contributes to beiging of white adipocytes, which leads to protection against obesity and the related disease T2D via heat generation and energy consumption. ILC2: group 2 innate lymphoid cells; MetEnk: methionine-enkephalin peptide; Mø: macrophages; T2D: type 2 diabetes mellitus.

**Table 1:** Pathophysiological roles of IL-1 family members. The cellular source, target cells, and major functions of IL-1 family cytokines are shown. Representative diseases involving each cytokine are also shown. The precise disease relevancy is described in the text.

Cytokine	Cellular sources	Targets	Major functions	Liver diseases	Other diseases
IL-1*α*	Many cell types	Many cell types	Inflammation	Sterile inflammation	Sterile inflammation

IL-1*β*	Many cell types	Many cell types	Inflammation, Th17 induction	Sterile inflammation NAFLD	Sterile inflammation Autoinflammatory diseases

IL-33	Many cell types	ICL2, basophils, eosinophils, mast cells	Wound healing Type 2 response	Liver fibrosis	Nematode-associated disease, allergic diseases, fibrosis

IL-18	Many cell types	NK cells, Th1 cells, mast cells, basophils	Inflammation, Th1 response, allergic response	Innate immunity-mediated liver injury	Chronic dermatitis, intrinsic allergy

IL-37	Unknown murine orthologue	Many cell types	Inhibition of innate and acquired immunity	Unknown	Protection of sepsis

IL-36	Many cell types	Skin	Recruitment of neutrophils	Unknown	Psoriasis

COPD: chronic obstructive pulmonary disease; ICL2: group 2 innate lymphoid cells; NAFLD: nonalcoholic fatty liver disease.

## Abbreviations

AID: Activation-induced cytidine deaminase
AIM2: Absent in melanoma 2
ALD: Alcoholic liver disease
APAP: Acetaminophen
ASC: Apoptosis-associated speck-like protein containing a carboxy-terminal CARD
BAT: Brown adipose tissue
B6: C57BL/6
CARD: Caspase recruitment domain
CCl_4_: Carbon tetrachloride
COPD: Chronic obstructive pulmonary disease
DAMPs: Damage-associated molecular patterns
DSS: Dextran sulfate sodium
FFA: Free fatty acid
HBV: Hepatitis B virus
HFD: High-fat diet
ICE: IL-1*β*-converting enzyme
IFN: Interferon
IGIF: Interferon inducing factor
IL: Interleukin
IL-18BP: IL-18-binding protein
IL-1Ra: IL-1R antagonist
ILC: Innate lymphoid cells
ILC2: Group 2 ILC
JNK: c-Jun-N-terminal kinase
MAPK: Mitogen-activated protein kinase
LPS: Lipopolysaccharide
LRR: Leucine-rich repeat
MAVS: Mitochondrial antiviral-signaling protein
MLKL: Mixed lineage kinase like
Mø: Macrophages
NF-*κ*B: Nuclear factor-*κ*B
NLR: Nod-like receptor
NLRC: NACHT, LRR, and CARD-containing protein
NLRP: NACHT, LRR, and PYD-containing protein
Pro: Precursor
PAMPs: Pathogen-associated molecular patterns
PYD: Pyrin domain
PBMC: Peripheral blood mononuclear cells
PCSK1: Proprotein convertase subtilisin/kexin type 1
Penk: Proenkephalin A
PR3: Proteinase 3
PRR: Pattern recognition receptor
PSTPIP2: Proline serine-threonine phosphatase interacting protein 2
R: Receptor
RIP: Receptor interacting protein kinase
RLR: RIGI-like receptor
RPP: Rhesus rotavirus type A
SAT: Subcutaneous WAT
SIGIRR: Single immunoglobulin IL-1-related receptor
Th: T helper
TIR: TLR/IL-1R
TLR: Toll-like receptor
TRIF: TIR-domain-containing adapter-inducing interferon-*β*
TRAIL: TNF-related apoptosis-inducing ligand
Treg: Regulatory T cells
UCP1: Uncoupling protein 1
VAT: Visceral adipose tissue
WAT: White adipose tissue
WT: Wild-type.
